# Challenges of caregiving to neurological patients

**DOI:** 10.1007/s10354-021-00844-8

**Published:** 2021-05-05

**Authors:** Gerhard Ransmayr

**Affiliations:** Department of Neurology II, MedCampus III, Kepler University Hospital, Johannes Kepler University, Medical Faculty, Krankenhausstraße 9, 4020 Linz, Austria

**Keywords:** Neurological diagnosis, Activities of daily living, Care, Behavioral impairment, Caregiver burden, COVID-19, Neurologische Diagnose, Alltagsaktivitäten, Pflege, Verhaltensstörungen, Betreuungsbelastung, COVID-19

## Abstract

A substantial number of neurological diseases lead to chronic impairment of activities of daily living (ADL) and physical or mental dependence. In Austria, homecare is provided mostly by female family members. Moreover, mainly female personnel, in the majority from southern and eastern European countries, contributes to care. Dependence and need for care vary between neurological diagnoses and accompanying diseases. Caregiver burden (CB) depends on patient- and caregiver-related and external factors, such as integrity of a family network, spatial resources, and socioeconomic factors. Depending on the neurological diagnosis, disease severity, and behavioral impairment and psychiatric symptoms, caregivers (CG) are at a significant risk of mental and somatic health problems because of limitations in personal needs, occupational and social obligations, financial burden, and restricted family life and leisure. Subjective and objective CB needs to be assessed in time and support should be provided on an individual basis. Recently, COVID-19 has caused additional multifactorial distress to dependent patients and informal and professional CG.

## Abstract (de)

Zahlreiche neurologische Erkrankungen führen zu chronischen Einschränkungen bei Alltagsaktivitäten und zu körperlicher sowie psychischer Abhängigkeit. In Österreich erfolgt die Betreuung meist durch Familienmitglieder, vor allem Frauen, aber auch durch meist weibliche professionelle Betreuungspersonen, überwiegend aus ost- und südeuropäischen Ländern. Die Art der Betreuung hängt primär von den neurologischen Diagnosen und Begleiterkrankungen ab. Die Betreuungsbelastung wird bestimmt durch von PatientInnen und von der Betreuungsperson abhängige und externe Faktoren, wie intaktes familiäres Netzwerk, Raumressourcen, sozioökonomische Bedingungen. In Abhängigkeit von der Diagnose, dem Schweregrad der Erkrankung sowie psychischen Symptomen und Verhaltensstörungen besteht für Betreuungspersonen ein signifikantes Risiko für psychische und physische Erkrankungen, bedingt durch Einschränkungen persönlicher Bedürfnisse, berufliche und soziale Verpflichtungen, Einschränkungen des Familienlebens und der Freizeit sowie finanzielle Belastungen. Subjektive und objektive Betreuungsbelastungen müssen rechtzeitig erkannt und umfassende Unterstützung auf individueller Basis angeboten werden. Neuerdings verursachte die COVID-19-Pandemie zusätzliche multifaktorielle Erschwernisse für informelle und professionelle Betreuungspersonen pflegebedürftiger Personen.

## Introduction

A substantial number of neurological diseases lead to chronic impairment of activities of daily living (ADL) and dependence. Neurological patients are cared for in their homes if impairments (motor, sensory, vegetative, cognitive, neuropsychiatric, or behavioral deficits) are manageable and if homecare is not precluded by spatial or socioeconomic limitations and support is sufficiently provided by family members alone or in cooperation with professional care persons. Otherwise, dependent patients move to the homes of family members or nursing homes. A need for professional care may occur unexpectedly as a consequence of significant deterioration of neurological symptoms or steadily increasing or acute failure of family-based homecare. In this context, concomitant or newly emerging non-neurological disorders and injuries of the patient and health problems of the caregiver (CG) may play a crucial role.

Caregiving is mostly time and energy consuming and a major challenge for CGs. Most CGs are female family members [1]. In 1986, caregiver burden (CB) related to the care of dementia patients was addressed for the first time in the medical literature [2]. Several hundred publications dealing with CB have been listed in PubMed thus far (https://www.ncbi.nlm.nih.gov/pmc/). In the past 20 years the number of publications has increased exponentially. CB has been addressed in the scientific literature from many different countries, cultures, and from all continents. The majority of the studies are monocentric and cross-sectional, but a substantial number of studies are longitudinal and multicentric. Some studies are transnational and cover a wider spectrum of caregiving-related issues. Interestingly, international comparative studies reveal differences between nations in the subjective and objective burden of care for patients with comparable diagnoses, disease severity, and age, probably due to differences in the workload of CGs, traditions, and medical, psychological, or financial support [1, 3, 4].

This article summarizes and reviews important issues of caregiving and CB in different neurological diagnoses and addresses recent challenges for patients and CGs during the COVID-19 pandemic, which have also been addressed in public media and websites of ministries and health and care organizations (www.sozialministerium.at; www.hilfswerk.at; www.volkshilfe.at).

## Results

### Neurological diagnoses leading to variable degrees and duration of dependence, need for care, and risk of caregiver burden

Neurological disorders prevail in old age, but may also manifest during childhood, adolescence, young adult age, or in midlife, such as multiple sclerosis, cerebral and spinal neoplasms, Huntington’s disease, traumatic brain injury, and neuromuscular and chronic pain disorders. Parkinsonian syndromes, stroke, and dementia usually occur in late life. Onset, course, degree, and type of dependence and need for care, and thus CB, differ between diagnoses, age at disease onset, sex, and the presence of dementia and behavioral abnormalities [5]. CB mainly depends on patient- and diagnosis-related factors; moreover, on educational and socioeconomic factors of the patient, the relationship between the client and the CG, professional and financial support, and the presence of a network of family members who contribute to care. CG-related factors of CB, such as age, sex, health status, and education are often underestimated or neglected [6, 7]. CB is mainly subjective; however, objective criteria of CB exist, such as daily duration of care, quality of sleep at night, psychological and somatic input, body mass index of the patient, degree of dependence, severity of cognitive and behavioral impairments, socioeconomic aspects, and support by other persons, etc. Both objective and subjective criteria of CB should be assessed systematically and incorporated in future concepts of public allowances.

1. Dementing illnesses are among the most frequent and devastating diseases in old age. The prevalence of dementia increases exponentially from the age of around sixty to very old age. Thirty to 50% of very old individuals suffer from significant neurocognitive disorders including dementia, caused by neurodegeneration, stroke, chronic vascular and metabolic disorders of the brain, neuroinflammation, (repeated) traumatic brain injury, toxic substances, medication, or a combination of these etiologies [4]. Depending on etiology, age at dementia onset, dementia severity, and comorbidities, dementia patients live maximum of 8–12, a mean of around 5 years after diagnosis. Male sex, old age, and behavioral, neuropsychiatric, and motor impairment are adverse prognostic factors [5–7]. Mortality is around 80% higher than in persons without dementia ([8, 9]; https://www.dementiacarecentral.com/aboutdementia/life-expectancy-calculator)
  Most dementias start insidiously and progress relentlessly. Thus, in most patients the need for care develops slowly, except in the case where a severe secondary disorder occurs. Daily care time and care intensity depend on etiology, disease duration, and severity. Medical treatment may achieve modest temporary improvement of cognitive symptoms, dependence, and CB [10, 11]. Neuropsychiatric symptoms and behavioral deficits in dementia accumulate mainly in the mid and late stages of dementias. They are important factors of CB and also prognostic factors of dementia [5, 7], but can be modified by pharmacotherapy and psychoeducative measures [12]. Therefore, medical and psychological support are important.
2. Parkinson’s disease and atypical parkinsonian syndromes are characterized by impairment of mobility, cognitive deterioration, neuropsychiatric and behavioral symptoms, and depending on the diagnoses (Parkinson’s disease, multiple system atrophy, dementia with Lewy bodies), also significant impairments of autonomic nervous system functions (orthostatic hypotension, collapse, with and without injuries, incontinence) and sleep [13–16]. Dependence and CB deteriorate with disease progression. Life expectancy is mildly reduced in Parkinson’s disease, markedly reduced in atypical parkinsonian syndromes (progressive supranuclear palsy, multiple system atrophy, corticobasal syndrome, and dementia with Lewy bodies) [17–20]. Mainly in atypical parkinsonian syndromes, the need for care starts early, especially in elderly persons, and is a major life-long challenge for CGs because of the complexity of symptoms and impairments.
3. In contrast to diseases summarized in the previous two paragraphs, a number of neurological disorders, such as stroke, traumatic brain injury, Guillain–Barré syndrome, or epilepsy, occur suddenly and may cause an acute need for care or observational accompaniment, which may cause major distress for patients and caregiving family members [21]. Neuro- and psychopharmacological treatment may be effective in avoiding or reducing dependence [22]. A crucial factor for partial, subtotal, or full functional recovery from functional deficits is comprehensive neurorehabilitation [23]. In Austria, even in severely disabled patients, acute care often needs to be taken over by family members, depending on administrative issues or waiting lists for free capacities in rehabilitation centers or nursing homes. Mainly in monophasic disorders such as stroke, CB may improve, mostly within weeks to months. Many patients, however, need permanent care for the rest of their lives. In epilepsies, often safety measures and surveillance rather than permanent care are needed.
4. Malignant brain tumor or motor neuron disease (MND) often develop subacutely and progress rapidly. In MND motor impairments prevail among other neurological symptoms. Life expectancy is mean 1 to 3 years [24, 25]. Most patients with these diagnoses are cared for by their families in their homes, often under difficult circumstances and major distress, supported by ambulatory palliative care. In motor neuron disease, mechanical ventilatory assistance may be needed. A variable proportion of motor neuron disease and brain tumor patients may suffer from intellectual, neuropsychological, and behavioral decline and communication difficulties [25–27]. Brain tumors are frequently associated with epileptic seizures.
5. Patients with chronic pain, such as neuropathic pain, due to acute, subacute, or chronic lesions of the central or peripheral nervous system may depend on care if the underlying disorder causes substantial impairment of ADL or technical support is needed, such as chronic parenteral analgesic therapies [28].

### Common features and factors of care burden

In most societies, care is mainly provided by family members—partners, daughters or daughters-in law, sons, and also parents, siblings, or more distant relatives or friends [1–4, 7]—mostly by women and mostly in the homes of the patients or in the household of the CG as long as care is manageable [29]. Apart from specific diagnoses, old age and male sex, body weight and height, impairment of mobility and impairments of ADL, dependence, cognitive decline, and neuropsychiatric and behavioral symptoms are important factors of CB [1–4, 7]. Frontal dysexecutive disorders (loss of insight, reasoning, logical thinking, etc.), behavioral impairment (hyperactivity or apathy, aggression, disinhibition), and neuropsychiatric symptoms such as irritability, anxiety, or depression, paranoic ideation and hallucinations, and communication difficulties are important factors of CB [5, 7, 25, 26, 29–32]. They may attenuate in late phases of dementing illnesses. Neuropsychiatric symptoms are frequent in dementias, mainly in dementia related to Parkinson’s disease, dementia with Lewy bodies, atypical parkinsonian syndromes (corticobasal syndrome and progressive supranuclear palsy), and frontotemporal dementia. Neuropsychiatric symptoms may vary and occur abruptly. Pharmacotherapy and psychoeducative measures are helpful to improve neuropsychiatric symptoms, especially in acute phases of neuropsychiatric deterioration. Nevertheless, CGs of patients with intermittent severe neuropsychiatric diagnoses often need immediate comprehensive (multidisciplinary) support.

Recent studies driven by the COVID-19 pandemic have shown that video consultations are beneficial for patients and CGs. For technical reasons, teleradiological consultations including video-visits may be challenging for elderly CG. However, the acceptance of video-visits was found to be high [33]. Strainful transfers to medical services can be minimized and patients and CGs can communicate with their doctors, psychologist, physiotherapist, nurse, or social worker in their usual familiar environment. Video-visit-based home rehabilitation programs have been developed. Video-visits may be less favorable for patients with severe impairments or symptom fluctuations than for patients with stable disease [33]. In summary, video consultations save time and seem altogether helpful to attenuate CB.

In late-stage neurological disease, falls, fluctuations of motor functions, complex therapies, repeated injuries, pain, fatigue, nausea, respiratory distress, incontinence, nocturnal sleep impairment, and concomitant diseases markedly deteriorate quality of life and exacerbate CB [14, 30–32, 34].

CB does not only depend on the patient, but also on CG-related factors such as age, sex, health status, quality of sleep, education, occupational obligations versus retirement, information about perspectives of the disease and medical, social, psychological, or legal consequences, personal competences, and supportive measures of the public health systems or private care institutions. Family background, relationship between the CG and the client, space in the patient’s home, socioeconomic parameters, and resilience of the CG [7, 31, 32, 34–37] are further important factors of CB. In most diseases CB increases with disease duration and progression, and with the duration of care. However, CB may stabilize with duration of care following adequate comprehensive support measures and in end stages of dementing illnesses. Knowledge about the individual needs of the patient and the CG as well as personal, psychological, and medical support may help the CG to cope with daily challenges of caregiving and improve the CG’s quality of life.

### Scales to assess care burden and underlying factors

In most studies analyzing CB in neurological diseases, inventories such as the Zarit CG Burden Interview (ZCBI), the CG Strain Index (CSI), the Burden Scale of Family CGs (BSFC), and patient- or CG-related quality of life interviews are used [38–41]. Objective measures of CB are less frequently applied, such as scales assessing informal care time [42]. Most of these inventories address specific questions about aspects and consequences of care and factors underlying CB, resulting in sum scores reflecting severity classes of CB (mild, moderate, severe CB) [38]. In some of the inventories, such as the ZCBI and the CSI, cut-off scores indicate thresholds to severe CB and a high risk of mental health problems for the CG [38, 39, 42]. CGs of neurological patients often report limitations in personal needs, privacy, self-determination (especially if patient and CG live together), strain with regard to occupation, family and social life, negative expectations and concern about the patient’s future, exhaustion, sleep impairment, and somatic and mental health problems. Moreover, financial distress, information deficits, a feeling of guilt and incompetence in providing appropriate care, of being left alone with the client, and negative emotions (irritation, anger) are reported [7, 26, 42]. Absence of support by other persons, low levels of education and income, living together with the patient, lack of leisure and recreation, and impaired health of the CG (psychiatric and somatic diagnoses) are determinants of CG-related CB. Caregiving children may be more burdened than partners because of unmet needs of caregiving, occupation, and family, conflicts of interests, and loss of contacts with friends [7]. CGs often give up their job or reduce working time, resulting in a loss of income.

### Comparison of severity of CB in neurological diseases

In stroke patients, patients suffering from a traumatic injury to the nervous system, and brain tumor patients, CB depends on the severity of functional decline including neuropsychiatric and behavioral deficits and the coherence of caregiving persons. CB is mostly severe in advanced stages of motor neuron disease and brain tumor disease, similar or somewhat milder in atypical parkinsonian syndromes [14, 24–26, 32]. Parkinson’s disease is associated with substantial CB in advanced but not in early disease stages, early Alzheimer’s disease with mild to moderate CB [7, 14, 25, 26] in the first years of the disease. Nevertheless, a substantial proportion of CGs of Alzheimer patients have a high risk of mental health problems, such as depression and burnout [7].

In multiple sclerosis patients, motor, vegetative (incontinence), and neuropsychological deficits, dependence, and need for care determine the severity of CB depending on the degree of functional deficits [42]. In stroke patients, CB depends on motor and sensory impairment, mobility, ADL, anxiety, and depression [43, 44].

### Which measures should be taken to prevent or to reduce care burden?

Studies have shown that support from other family members or formal (professional) CGs are prerequisites for a CG to take a break, to have time for personal needs such as leisure, recreation, sports, social contacts, occupation, and family. Networks of dementia-specific care institutions are very helpful in supporting caregiving family members (such as www.alzheimer-hilfe.at). Holidays for pairs (partners of dementia patients together with the demented client, supported by professional CGs) are highly appreciated. CGs feel significantly better when caregiving is appreciated by the client, the family, and also the public [45]. CB is lower in families with close ties between family members, personal support, respect, and understanding of the CG’s needs. In pauses from care for personal needs or obligations, family CGs need confidence that their loved one is competently cared for by other persons [46]. A health psychologist should be contacted if CGs suffer from emotional distress. Financial support is an important issue, in particular for persons with low income, for compensation of financial strain, and personal support. Exchange of personal experiences between CGs in self-help organizations and specific information may also attenuate CB.

In our country (Austria), public allowances for caregiving (*Pflegegeld*) are mainly based on the average time needed for care per week (www.oesterreich.gv.at). In demented persons, care is considered more demanding than in clients with other diagnoses, so that the allowance rates for dementia care are higher than in other diagnoses. Nevertheless, important factors contributing to subjective and objective CB and strain, such as behavioral abnormalities, severity, and nature of dependence as well as CG-related factors and the burden for the families are not specifically reflected in the funding procedures. A more comprehensive assessment of objective and subjective CB is required in time, to take adequate support measures, to maintain high-quality care, and to prevent the CG from severe CB and health problems.

### Caregiving and the COVID-19 pandemic

Caregiving for senior residents in our country (Austria) relies on informal family members and on mostly foreign, mainly female, professional CGs. Around 60,000 foreign professional CGs work in Austrian households (www.ooe.arbeiterkammer.at; www.derstandard.at, www.ams.at). Both informal and formal CGs and patients, as reported in detail for dementia patients, were struck by the COVID-19 pandemic [47]. Travel restrictions resulted in deficits of professional CGs for Austrian patients. Instead, family members often take over care functions. Many of them also had to cope with homeschooling and homeoffice. For those persons, caregiving became extremely challenging. Extra nursing leave was legalized. Foreign CGs who could not return to their families remained in Austria for longer periods of time than primarily intended. To compensate for this extra work, public allowances were provided by the government. CB also increased because of hygiene measures, such as the mouth/nose mask, which is problematic for patients who forget the meaning of this measure and persons with senile hearing impairment, communication deficits, or respiratory illness. Physical and social distancing including family members in nursing homes has caused major psychological distress in patients and therefore also in CGs (www.tnp.sg/lifestyle/health/managing-caregiver-stress-during-covid-19). Before vaccination became available, CGs involved in the care for COVID-19-infected persons were at a high risk of becoming infected. In summary, the COVID-19 pandemic is a major general stressor for CGs working at home and in nursing homes in general, and also for neurological patients.

## Conclusion

CB is a largely underestimated issue and needs more political and public awareness. There is no systematic and standardized tool to assess overall (objective and subjective) CB from the start of a care. These factors of CB need to be assessed as early as possible to provide individualized support and to avoid a collapse of homecare and premature admission to a nursing home. The network of multidisciplinary support needs improvement and all possible resources need to be integrated. Financial support (public allowances) is often delayed or even rejected. The need for professional care increases steadily. More professional CGs are needed, as is specific training for CGs of patients with neurological diagnoses, in order to provide the best possible care for clients with neurological diseases.
